# Could the Urease of the Gut Bacterium *Proteus mirabilis* Play a Role in the Altered Gut–Brain Talk Associated with Parkinson’s Disease?

**DOI:** 10.3390/microorganisms11082042

**Published:** 2023-08-09

**Authors:** Matheus V. C. Grahl, Brenda da Silva Andrade, Ana Paula A. Perin, Gilda A. Neves, Laura de Souza Duarte, Augusto Frantz Uberti, Kelvin Siqueira Hohl, Cristian Follmer, Celia Regina Carlini

**Affiliations:** 1 Graduate Program in Medicine and Health Sciences and Brain Institute, Pontifical Catholic University of Rio Grande do Sul, Porto Alegre 90610-000, RS, Brazil; matheusgrahl@hotmail.com (M.V.C.G.); afuberti@gmail.com (A.F.U.); 2 School of Health Sciences, University Center Ritter dos Reis, Porto Alegre 90840-440, RS, Brazil; 3 Laboratory of Molecular Pharmacology, Institute of Biomedical Sciences, Health Sciences Center, Federal University of Rio de Janeiro, Rio de Janeiro 21944-590, RJ, Brazil; brendasilvaandrade21@gmail.com (B.d.S.A.); ganeves@icb.ufrj.br (G.A.N.); duarte.laura.rj@gmail.com (L.d.S.D.); 4 Graduate Program in Cellular and Molecular Biology, Center of Biotechnology, Federal University of Rio Grande do Sul, Porto Alegre 91501-970, RS, Brazil; anaperin.app@gmail.com; 5 Graduate Program in Biological Sciences—Biochemistry, Federal University of Rio Grande do Sul, Porto Alegre 90035-003, RS, Brazil; kelvin.hohl@outlook.com; 6 Laboratory of Biological Chemistry of Neurodegenerative Disorders, Institute of Chemistry, Department of Physical-Chemistry, Federal University of Rio de Janeiro, Rio de Janeiro 21941-909, RJ, Brazil; crisfollmer@gmail.com; 7 Department of Biochemistry, Federal University of Rio Grande do Sul, Porto Alegre 90035-003, RS, Brazil; 8 National Institute of Science and Technology in Brain Diseases, Excitotoxity and Neuroprotection (INCT-EN), Porto Alegre 90035-003, RS, Brazil

**Keywords:** neuroinflammation, α-synucleinopathies, Parkinson’s disease, bacterial ureases, behavioral analysis, motor deficits

## Abstract

Intestinal dysbiosis seems to play a role in neurodegenerative pathologies. Parkinson’s disease (PD) patients have an altered gut microbiota. Moreover, mice treated orally with the gut microbe *Proteus mirabilis* developed Parkinson’s-like symptoms. Here, the possible involvement of *P. mirabilis* urease (PMU) and its B subunit (PmUreβ) in the pathogenesis of PD was assessed. Purified proteins were given to mice intraperitoneally (20 μg/animal/day) for one week. Behavioral tests were conducted, and brain homogenates of the treated animals were subjected to immunoassays. After treatment with PMU, the levels of TNF-α and IL-1β were measured in Caco2 cells and cellular permeability was assayed in Hek 293. The proteins were incubated in vitro with α-synuclein and examined via transmission electron microscopy. Our results showed that PMU treatment induced depressive-like behavior in mice. No motor deficits were observed. The brain homogenates had an increased content of caspase-9, while the levels of α-synuclein and tyrosine hydroxylase decreased. PMU increased the pro-inflammatory cytokines and altered the cellular permeability in cultured cells. The urease, but not the PmUreβ, altered the morphology of α-synuclein aggregates in vitro, forming fragmented aggregates. We concluded that PMU promotes pro-inflammatory effects in cultured cells. In vivo, PMU induces neuroinflammation and a depressive-like phenotype compatible with the first stages of PD development.

## 1. Introduction

Ureases (EC 3.1.3.5, urea amidohydrolase) are enzymes that catalyze the hydrolysis of urea into carbon dioxide and ammonia. These proteins are synthesized by plants, fungi, bacteria, and archaeans, but they are not produced by animals [1]. Regardless of the origin, ureases share at least ~55% identity across the phyla and may contain one, two, or three subunits organized in complex quaternary structures [2]. 

*Proteus mirabilis* is a Gram-negative and urease-positive bacterium, naturally found in the intestinal tract [3]. *P. mirabilis* urease (PMU) displays three types of subunits, A or γ, B or β, and C or α, and it occurs as a trimer of trimers (αβγ)_3_ [4]. An opportunistic uropathogen, *P. mirabilis* causes ~10% of all urinary tract infections (UTI) (cystitis, pyelonephritis, urolithiasis, and prostatitis) [5,6]. Moreover, *P. mirabilis* causes other types of pathologies, such as ophthalmological [7] and respiratory [8] infections, empyema [9], osteomyelitis [10] and neonatal meningitis [11,12]. Studies by our group indicated that PMU might contribute to the pathogenicity of *P. mirabilis* by inducing deleterious effects independent of its enzymatic action [13]. We have demonstrated that PMU has platelet-aggregating activity and increases the levels of pro-inflammatory cytokines (TNF-α and IL-1β) in cultured human renal cells (Hek 293) and in murine microglia (BV-2). We also observed that PMU could induce the production of reactive oxygen species in Hek 293 and SH-SY5Y (human neuroblastoma) cells [13]. 

Neurodegenerative diseases are caused by the progressive loss of certain populations of neurons, causing cognitive, physical, and motor symptoms [14]. The progressive death of neurons can originate from several factors, such as proteotoxic stress, alterations in the ubiquitin–proteasome system, deficits in phagosome–lysosome activity, oxidative stress, and neuroinflammation [14]. Neurodegenerative diseases can be hereditary or caused by unknow factors that lead to changes in the form or state of aggregation of some pivotal proteins (tauopathies, α-synucleinopathies, and amyloidosis) [15,16]. 

Parkinson’s disease (PD) is the second most prevalent neurodegenerative disorder. PD is characterized by motor deficits (tremors, muscle stiffness, and postural abnormalities) as well as by non-motor symptoms (cardiovascular, urological, olfactory, and gastrointestinal) [17]. Although some genetic causes of PD are known, most cases are sporadic, with unknown pathogenesis. The histopathological hallmarks of PD are the presence of Lewy bodies (accumulation of aggregated α-synuclein) and death of dopaminergic neurons [18,19]. In some cases, aggregates of α-synuclein originate first in the enteric nervous system (ENS) and spread from there in a prion-like fashion via the vagus nerve into the central nervous system (CNS) [20,21,22]. In sporadic PD, it has been proposed that a putative environmental pathogen (or exotoxin) capable of crossing the gastric/intestinal epithelial lining could induce misfolding and aggregation of α-synuclein in a subset of cells of the submucosal plexus and reach the brain following a consecutive series of projection neurons [19,23,24,25]. 

A profound alteration in the gut microbiota has been described in PD patients, with the prevalence of *Enterobacteriaceae* bacteria, among which is *P. mirabilis*, abundantly present in the stool [26,27]. Changes in the permeability of the intestinal epithelial barrier and/or the blood–brain barrier, resulting from the action of pro-inflammatory molecules, toxins, or metabolites produced by pathogens, potentially facilitate migration of active compounds from the bloodstream into the CNS [28,29]. In the context of intestinal dysbiosis, the increase in cytokines or interleukins and accumulation of anomalous protein aggregates, such as those of α-synuclein observed in the ENS, can further spread to the CNS [28,29]. In agreement with an ENS origin, complete vagotomy in PD patients decreased the disease’s progression when compared to those who did not undergo or had a partial procedure [19,30,31,32]. 

Aiming to establish the contribution of *P. mirabilis* to the pathogenesis of PD, Choi et al. (2018) treated mice orally with a suspension of live bacteria [18]. The animals developed PD-like motor symptoms and a loss of dopaminergic neurons in the substantia nigra and striatum, accompanied by increased levels of pro-inflammatory markers (Iba1, TNF-α, and IL-1β) and α-synuclein deposition in the brain and colon [18]. *P. mirabilis* produces several virulence factors, including the enzyme urease, which by catalyzing the hydrolysis of urea from urine, generates ammonia and contributes to the formation of urinary stones. These, in turn, enclose the bacteria and protect them against antibiotic therapy, allowing the pathogen to survive and proliferate [5,33]. 

*Helicobacter pylori* is another urease-positive bacterium associated with neurodegenerative diseases. This bacterium colonizes the gastric mucosa, causing an inflammatory response with an increase in pro-inflammatory cytokines (IL-1β, IL-6, IL-8, and TNF-α) [34]. *H. pylori* is also associated with extra-gastric pathologies, such as cerebrovascular [35] and vascular diseases [36], mild cognitive impairment [37], PD [38] and Alzheimer’s disease [39,40,41]. Production of urease is an absolute requirement for gastric colonization by *H. pylori*, as the ammonia generated by the enzyme alkalizes the stomach mucosa, allowing the pathogen’s survival [42]. We have shown that *H. pylori* urease (HPU) can induce several non-enzymatic effects, such as the activation of blood platelets with conversion into a pro-inflammatory phenotype [43,44], activation of neutrophils [45], production of NO, reactive oxygen species, pro-inflammatory cytokines, and increased paracellular permeability in endothelial cells [46,47]. In cultures of SH-SY5Y and BV-2 (CNS-derived) cells, HPU triggered the production of reactive oxygen species and pro-inflammatory cytokines (TNF-α and IL-1β). Animals treated with HPU i.p. for 5 consecutive days showed no behavioral changes. However, elevated levels of microglial activation and tau protein phosphorylation were observed in brain homogenates of HPU-treated rats, consistent with neuroinflammation [46].

The ammonia-independent toxicity of ureases was first described for canatoxin [48], a neurotoxic urease isolated from *Canavalia ensiformis* (jack bean), which maintained its biological effects even after its ureolytic active site was irreversibly blocked [49]. Following these findings, our group has described the non-ureolytic effects of ureases from different sources, including the bacterial ureases from *Sporosarcina pasteurii*, *H. pylori* (reviewed in [1,2]) and *P. mirabilis* [13,50]. Among these enzyme-independent effects of ureases are platelet aggregation [44,51,52], chemotaxis induction [45,53], exocytosis [44,54], production of reactive oxygen species [13,45], neurotransmitter release [54,55], production of pro-inflammatory cytokines [13,43,46,47] and changes in Ca^2+^ transport [13,46,52,54]. In the case of *P. mirabilis* urease (PMU), the pro-inflammatory effects in cultured cells observed for the holourease (αβγ)_3_ were not associated with an increase in the ammonia levels in the medium, discarding the contribution of the enzyme activity [13]. In another study, the isolated subunits of PMU were tested, revealing that the β subunit (PmUreβ) alone carries most of the non-enzymatic properties of the protein [50].

Here, we aimed to investigate the biological properties of PMU that could be relevant to the pathogenesis of Parkinson’s disease. For that goal, the purified holoenzyme was tested in cultured cells and administered to mice. Homogenates of cell cultures or brain tissues were analyzed for cytotoxic and pro-inflammatory effects. The treated mice were evaluated for behavioral alterations following the protocol of Choi et al. (2018) [18]. Aiming to elucidate the structure versus activity relationships for PMU, tests were also conducted with its isolated β-subunit. Finally, the formation of α-synuclein fibrils was investigated in vitro in the presence of PMU, PmUreβ, and the *H. pylori* urease (HPU). 

## 2. Materials and Methods

### 2.1. Purifications

#### 2.1.1. Holoenzyme–PMU

Production of the recombinant *P. mirabilis* urease was performed in *E. coli* according to Grahl et al. (2021) with modifications [13]. Chromatographies were performed on an ÄKTA^TM^ apparatus (GE Healthcare, Little Chalfont, UK), and fractions from all the steps were analyzed for ureolytic activity and via 15% SDS-PAGE. Briefly, the cell extract was applied to a HiPrep Q XL 16/10 (GE Healthcare, Little Chalfont, UK) equilibrated in 20 mM sodium phosphate buffer (NaPB), pH 7.0, and washed with the same buffer to remove unbound proteins. The ureolytic activity was eluted between 0.3 and 0.4 M KCl in NaPB 7.0, in a 20 mL gradient, at a 3 mL·min^−1^ flow rate. The urease-enriched fractions were pooled and dialyzed against NaPB adjusted to pH 7.5 (NaPB 7.5), filtered on a 0.22 µm filter and then loaded into a Source^TM^ 15Q column (GE Healthcare, Little Chalfont, UK) equilibrated in NaPB 7.5. Elution was performed with a 10 mL linear gradient of KCl in NaPB 7.5, at a 1 mL·min^−1^ flow rate. The active fractions, eluted between 0.3 and 0.4 M KCl, were pooled and concentrated using a Vivaspin^TM^ (GE Healthcare, Little Chalfont, UK) device with a 100 kDa cut-off. This material was then gel-filtered on a Superdex 200^TM^ 26/60-pg (GE Healthcare, Little Chalfont, UK) and eluted in NaPB 7.0 containing 150 mM KCl (see Supplementary Figure S1). Finally, the pool of gel-filtered fractions was dialyzed against NaPB 7.5 and applied into a Source^TM^ 15Q column using the same conditions described above. The active fractions were pooled and designated as purified PMU. 

#### 2.1.2. PMU B Subunit–PmUreβ 

The recombinant PmUreβ was expressed and purified as described by Broll et al. (2021) [50]. Briefly, recombinant *E. coli* (Lemo21) cells carrying the PmUreβ sequence were multiplied in LB medium with 100 µg·mL^−1^ of ampicillin and 40 µg·mL^−1^ of chloramphenicol (Sigma-Aldrich, St. Louis, MO, USA). Cultures were performed at 37 °C under constant agitation (180 rpm). Protein expression was induced overnight at 18 °C with 0.3 mM IPTG and 100 µM rhamnose until the cellular growth achieved an OD600 of 0.7. The PmUreβ-containing cells were centrifuged at 6000× *g* for 10 min at 4 °C. The pellet was suspended in buffer containing 50 mM Tris-HCl pH 7.5, 500 mM NaCl and 20 mM imidazole. The cells were disrupted by sonication and the cellular debris were pelleted by centrifugation at 14,000× *g* for 30 min. PmUreβ was found in the culture supernatant. The recombinant subunit contained a His tag at the C-terminal portion. Purification via affinity chromatography was performed on a Chelating Sepharose column (GE Healthcare, Little Chalfont, UK) equilibrated in 50 mM Tris-HCl pH 7.5 buffer, 500 mM NaCl and 20 mM of imidazole. The column was washed with the same buffer containing 70 mM imidazole and then eluted with 500 mM of imidazole. Before each bioassay, a dialysis was conducted to change the buffer to 10 mM Tris-HCl pH 7.5 buffer and 1 mM DTT. 

#### 2.1.3. HPU

Recombinant *H. pylori* urease (HPU) was produced via heterologous expression in *E. coli* BL21 (DE3)-RIL transformed with a PGEM-T Easy (Promega, Madison, WI, USA) plasmid carrying the whole urease operon (kindly provided by Dr. Barbara Zambelli, Universitá di Bologna, Italy). The HPU was purified from bacterial extracts according to Wassermann et al. (2010), with the small modifications introduced in Scopel-Guerra et al. (2017) [43,44]. The protein purity was verified by means of SDS-PAGE.

### 2.2. Protein Determination and Sterilization

The protein contents were determined via absorbance at 280 nm or via the Bradford, (1976) method, using bovine serum albumin as the standard [56]. Solutions of all the proteins were sterilized by passing through 0.22 µm syringe filters before performing the biological assays.

### 2.3. In Vivo Assays

All the procedures were previously approved by local animal ethical committees (PUC/RS authorization 10910/2022; CEUA/UFRJ approval 142/19) and performed according to the Directive of the European Parliament and of the Council of the European Union (2010/63/EU) and the guidelines from the Brazilian National Council for Animal Experimentation Control (CONCEA). 

#### 2.3.1. Acute Effects and Toxicity Monitoring

Twenty-one male thirty-day-old Balb/C mice (~20 g/body weight), kept at 22 ± 3 °C with a 12/12 h light/dark cycle, were obtained from the Center for Biological Experimental Models (CeMBE-PUCRS, Porto Alegre, Brazil). The animals were injected endovenously (e.v.) in the tail vein with PMU at a 20 mg/kg dose or with sterile saline and monitored for 4 h for changes in their body temperature and blood glucose levels. Blood samples were obtained from small perforations in the tail tip, and the glucose levels were determined using a digital glucometer (Accu-Check, Roche Diabetes Care, Inc., Mannheim, Germany). After administration of PMU, the animals were monitored for 3 h with an interval of 1 h between each check [57]. The rectal temperature was measured in the Balb/C mice with a digital thermometer, for 1 min, at 1 h intervals up to 3 h [48].

After that, the animals were injected intraperitoneally (i.p.) with 2% Evans blue and followed for 3 or 24 h to detect blood–brain barrier disruption (see Section 2.4). In addition, five Balb/C male mice and five Wistar male rats (~45 days old) were treated (e.v.) with 20 mg/kg of PMU and kept in their cages, with water and food ad libitum, for the monitoring of toxicity signs. Finally, after 48 h of careful observation, the animals were anesthetized with thiopental (100 mg/kg, i.p.) and euthanized by decapitation.

#### 2.3.2. Treatments and Behavioral Analysis

To verify the possible role of *P. mirabilis* urease in the pathogenesis of Parkinson’s disease, we followed a similar protocol for behavioral and motor evaluation as proposed by Choi et al. (2018) [18]. Thus, 45 male Swiss mice (~55 days old) from the Institute of Biomedical Sciences (ICB-UFRJ, Rio de Janeiro, Brazil) breeding colony were used. The animals were kept at 23 ± 2 °C under a 12/12 h light–dark cycle (lights on at 6 a.m.) with ad libitum access to filtered water and standard chow. 

The mice were separated into 3 experimental groups (control, PMU, PmUreβ), with 15 animals in each. They received two days of training to perform the motor tests. After, the animals received daily (i.p.) injections of 20 µg/animal PMU or 20 µg/animal PmUreβ (dose ~1 mg protein/kg body weight), or buffer (10 mM Tris-HCl pH 7.5 buffer and 1 mM DTT) as the control, for 7 days. The behavioral tests were performed twice starting 7 days after the last injection: days 7 and 15—anxiety test and rotarod; days 8 and 16—open field and wire hanging box; days 9 and 17—pole test and tail suspension. After the experiment, the animal was given 1 h of recovery before the next test. On the 18th day, 30 animals were euthanized by decapitation, and their brains were collected. Moreover, 15 animals were injected with 2% Evans blue (0.01 mL/100 g body weight) and euthanized 24 h after for analysis of the blood–brain barrier integrity (see Section 2.4). Supplementary Figure S2 summarizes the experimental design. 

#### 2.3.3. Motor Tests

Three classical motor tests were selected to evaluate the potential motor impairments induced by PMU repeated administration. The rotarod and pole tests evaluate motor coordination, while the wire hanging box assesses mice’s neuromuscular strength. On the first experimental day, the animals were trained for the rotarod and wire hanging tests, and on the second day, for the rotarod and the pole test. Then, they were reevaluated 7–9 and 15–17 days after the treatments.

##### Rotarod

The device consisted of a 5 cm diameter cylinder that gradually increased its rotation from 5 to 16 rpm. The animals were placed on the rotating rod, and the speed was gradually increased, reaching its maximum after 5 min. Each test day consisted of three 5 min sessions, with an interval of 1 h between them. The integrity of the motor coordination was evaluated based on the animals’ average length of stay on the rod each day [58,59]. 

##### Wire Hanging Box

A transparent plexiglass box with a wire grid on its lid was used for this test. The animals were gently placed on top of the lid until they grasped the grid, and the lid was then slowly turned upside down. The grid was held ~20 cm above a soft base, high enough to prevent the mouse from jumping but that would not cause damage in case of a fall. The animal’s hanging time on the grid was measured in three attempts per day (5 min maximum), with an intertrial interval of 1 h. The daily average latency to fall was used to assess the mice’s performance [60].

##### Pole Test

The mice were gently placed facing upward on the top of a rough-surfaced vertical pole (diameter 8 mm; height 55 cm). The time taken for the animal to turn 180° and the latency for its four paws to touch the ground (locomotor activity time) were recorded. Every experimental session comprised three tests, with an intertrial interval of 1 h, and the average time per day was considered. When the animal could not turn toward the ground or fell from the pole, the time was recorded as 5 min (maximum) [59,61].

#### 2.3.4. Open Field

The open field task is used to evaluate the general motor behavior of rodents. Analysis of thigmotaxis is also usually performed to assess mice’s anxiety profile. For this test, the animals were placed in the center of a square arena (20 × 20 cm) under indirect light and left free to explore it for 10 min. Each mouse had its trajectory filmed. The total distance covered (cm), the percentage of the distance, and the time spent in the center of the box (10 × 10 cm) were calculated using the *MouseGlob* v. 1.0 open-source software [62].

#### 2.3.5. Anxiety Tests

##### Elevated Plus Maze

The plus maze consisted of two open arms (35 × 5 × 1 cm) and two enclosed arms (35 × 5 × 15 cm), 40 cm above the floor. The arms extended from a 5 × 5 cm central platform, arranged so that the two arms of each type were in opposite positions. The mice were placed in the center of the maze facing one of the open arms, and for 5 min, the following measurements were recorded: the number of entries, the time spent in the open and closed arms, and the number of risk assessments (number of times the animal entered the open arms with only two paws to explore and assess the risk of moving forward) [63]. The tests were conducted under low indirect light, and the mice’s performance was evaluated on day 7 post-treatments (Supplementary Figure S2).

##### Dark–Light Box

The mice were evaluated in this test 15 days after the treatments (Figure S2) to avoid repeating the same conflict-based anxiety test. The dark–light box measured 45 × 20 × 30 cm, divided into two compartments: a dark one (15 × 20 × 30cm) and a light one (400 lux, 15 × 20 × 30 cm), divided by a small gate. The mice were initially placed in the middle of the light compartment facing toward the gate, and they freely explored the box for 5 min. The sessions were recorded to allow the analysis of the number of transitions from the dark to the light side. The total traveled distance inside the box was calculated using the *MouseGlob* v. 1.0 open-source software [62], and the percentage of traveled distance in each compartment was subsequently calculated. The time spent in the light compartment is an inverse measure of anxiety-like behavior [64].

#### 2.3.6. Tail Suspension Test

The tail suspension test was performed to investigate the development of depressive-like behavior. In this test, mice tend to develop an immobile posture when placed in an inescapably stressful situation after initial escape movements. The longer the animal stays in this immobile posture, the higher its sensitivity to a stressful situation, a behavioral correlate of a depressive-like phenotype [65]. The mice were suspended individually (using an adhesive tape placed 1 cm from the tip of the tail) about 40 cm above a soft base for 6 min. The immobility time was recorded only when the mice were passively hanging completely still. The sessions were videotaped, and the immobility time(s) was blindly scored.

### 2.4. Integrity of the Blood–Brain Barrier

The permeability of the blood–brain barrier (BBB) was tested in the mice after the treatments (see Section 2.3.1 and Section 2.3.2) by injecting (i.p.) a 2% Evans blue solution (0.01 mL/100 g body weight). After 3 or 24 h following the Evans blue injection, the animals were anesthetized with thiopental (100 mg/kg, i.p.) and decapitated. Their brains were quickly dissected, with the two hemispheres being separated and homogenized in phosphate buffered saline (1100 µL/hemisphere). The homogenates were centrifuged for 30 min at 1500× *g* at 4 °C, the supernatants were mixed with 50% acetic acid 1:1 (*v*/*v*) and kept overnight at 4 °C. The samples were centrifuged again (30 min, 1500× *g*, 4 °C), and the absorbance of the supernatants was measured at 610 nm in an M2 spectrofluorometer [66].

### 2.5. Western Blot

#### 2.5.1. Preparation of Brain Homogenates

The mice whole brains were dissected and homogenized in lysis buffer [20 mM Tris-HCl pH 7.4, 1% NP-40, 1 mM EDTA, 1 mM EGTA, 1 mM PMSF, 1 mM Na_3_VO_4_, and a protease inhibitor cocktail (Sigma, St. Louis, MO, USA)] using a microtube pestle. The tissue homogenates were centrifuged at 12,000× *g* for 10 min at 4 °C, and the supernatants were stored at −80 °C [46].

#### 2.5.2. Western Blot Analyses

The brain homogenates were denatured in sample buffer (50 mM Tris-HCl, pH 6.8, 1% SDS, 5% 2-mercaptoethanol, 10% glycerol, 0.001% bromophenol blue) and heated in a dry bath for 5 min. The samples (20 µg of total protein) were separated on SDS-PAGE gels and the proteins were transferred onto 0.22 µm nitrocellulose membranes (BioRad, Hercules, CA, USA). The molecular mass markers (PageRuler, Thermo-Scientific, Waltham, MA, USA) were run in parallel. The membranes were blocked with PBS-Tween (137 mM NaCl, 2.7 mM KCl, 10 mM Na_2_HPO_4_, 1.8 mM KH_2_PO_4_, 0.1% Tween-20) containing 5% bovine serum albumin (Sigma) and subsequently incubated with the following antibodies: rabbit anti-alpha synuclein (Invitrogen, Carlsbad, CA, USA, PA517239, 1:1000), rabbit anti-tyrosine hydroxylase (Invitrogen, OPA104050, 1:1000), mouse anti-Tau46 (Cell Signaling, Danvers, MA, USA, #4019, 1:1000), rabbit anti-caspase9 (Cell Signaling, #9504, 1:1000), rabbit anti-Iba1 (Invitrogen, PA527436, 1:1000), rabbit anti-p-selectin (Santa Cruz Biotech, Santa Cruz, CA, USA, SC-8419, 1:1000), rabbit anti-pTau199 (Invitrogen, 701054, 1:1000), and rabbit anti-actin (Sigma-Aldrich, St. Louis, MO, USA, A2066, 1:1000). Secondary antibodies (anti-mouse and anti-rabbit, 1:10,000) coupled to horseradish peroxidase were obtained from Jackson ImmunoResearch Laboratories, Inc., West Grove, PA, USA). The protein bands were visualized using a chemiluminescence detection kit (Millipore, Billerica, MA, USA) with a L-Pix Chemi (Loccus, Cotia, Brazil) apparatus. The levels of protein expression were quantified using the software ImageJ v.8.0 and normalized against β-actin as an endogenous control [46].

### 2.6. Cytokine Detection

Caco-2 (human intestinal epithelium) cell cultures were incubated with NaPB 7.0 (control), 63, 126 or 252 nM PMU. After 6 h, the supernatants were collected and stored at −80 °C. The contents of IL-1β and TNF-α were determined via ELISA using commercial kits (Invitrogen, CA, USA, product code) according to the manufacturer’s instructions: murine IL-1β (88-7013-22) and murine TNF-α (88-7324-22).

### 2.7. Permeability of Cell Junction

Hek 293 (human embryonic kidney) cells were cultured (5 × 10^4^ cells/well) onto Transwell inserts (0.4 μm) for 72 h to form a confluent monolayer. Alterations in the epithelial monolayer permeability were evaluated by monitoring the passage of FITC-Dextran. The cells were stimulated with PMU (63, 126, 252 nM) or buffer (control) for 60 min. FITC-Dextran (1 mg/mL; Sigma-Aldrich) was then added onto the top of each insert, and after 30 min, 20 μL samples were collected from the bottom compartment. The fluorescence emitted by the FITC-Dextran (excitation and emission wavelengths of 495 and 530 nm, respectively) was quantified using an M2 spectrofluorometer (Molecular Devices, San Jose, CA, USA) and expressed as the relative fluorescence intensity [47]. 

### 2.8. Fibrillation of α-Synuclein 

Recombinant human α-synuclein was produced in BL21(DE3)pLysS and purified as previously described by Coelho-Cerqueira et al. (2013) [67]. 

In order to promote α-synuclein aggregation, 50 μM of recombinant wild-type protein was incubated with 5 μM of either PMU, PmUreβ or HPU in 10 mM sodium phosphate, pH 7.5, 100 mM NaCl. The control reaction contained only α-synuclein and buffer. The mixtures were kept under agitation (350 rpm) at 37 °C for 150 h. The morphology of the α-synuclein aggregates generated in the absence or in the presence of PMU, PmUreβ or HPU was characterized using negative-staining transmission electron microscopy (TEM) [68]. The samples were 5-fold diluted, and 10 μL of this material was deposited on to a Formvar/carbon 200 mesh copper grid (Ted Pella Inc., Redding, CA, USA). The samples were stained with 10 μL of uranyl acetate (2% aqueous solution) for 30 s, dried, and then examined under a JEOL 1200EX (National Center of Structural Biology and Bioimaging–CENABIO/UFRJ, Rio de Janeiro, Brazil). 

### 2.9. Statistical Analysis

All the results are expressed as the mean ± standard error of the mean (SEM). Student’s *t* test and one-way and two-way ANOVA for single and repeated measures were used. When appropriate, the Tukey post hoc test was also applied. The adequate hypothesis test for each dataset was selected considering the experimental design, the number of experimental groups, and the data distribution. Statistical significance was set at a *p*-value ≤ 0.05. GraphPad Prism 8.02 software (San Diego, CA, USA) was used to perform the statistical analysis.

## 3. Results

### 3.1. Acute In Vivo Effects of PMU

Administration of ureases to rodents can cause several systemic effects, such as bradycardia, hypoglycemia, hypothermia, hypoxia, and seizures, that eventually precede death [1,55]. Here, we aimed to evaluate the acute (within 24 h) in vivo effects triggered by systemic (e.v.) administration of a single dose of 400 μg of purified *P. mirabilis* urease per animal (20 mg/kg). 

**Figure 1:**
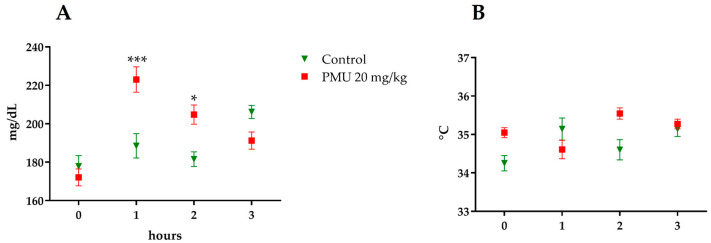
Acute in vivo effects. The mice were injected e.v. with PMU 20 mg/kg and monitored for 3 h. The blood glucose levels were determined with a blood glucometer (**A**). Body temperature monitoring was carried out using a rectal thermometer. (**B**). The data were analyzed via a two-way ANOVA of the row stats. The results are expressed as the mean ± SEM (n = 5 for the control, 10 for treatments). * *p* < 0.05 and *** *p* < 0.001.

The Balb/C mice treated with PMU showed a glycemic peak 1 h after administration that persisted for 2 h and returned to basal levels after 3 h (Figure 1A). No changes in the animals’ body temperature were seen after the toxin administration (Figure 1B). The Balb/C mice and Wistar rats had no seizures and survived at least 48 h after the endovenous administration of a single PMU dose (20 mg/kg).

### 3.2. Effects in Mice of 7-Day Treatment with Purified PMU or PmUreβ 

After that, we aimed to investigate whether PMU could be involved in the motor or behavioral changes presented by mice dosed orally with a *P. mirabilis* suspension, as described by Choi et al. (2018) [18]. Therefore, we exposed the mice daily to purified PMU via intraperitoneal injections. Furthermore, aiming to determine the structure versus activity of PMU and considering that it β subunit (PmUreβ) was shown to induce some of the biological effects of the whole urease [50], a group of animals was dosed with PmUreβ (20 μg protein/animal). The vehicle used to solubilize the proteins (10 mM Tris-HCl pH 7.5 buffer and 1 mM DTT) was used as the control.

#### 3.2.1. Motor Tests

The pole test, rotarod, and wire hanging box are used to assess motor impairment in mice models of movement disorders to evaluate changes in motor coordination, balance, and muscular endurance [61,69,70]. 

**Figure 2:**
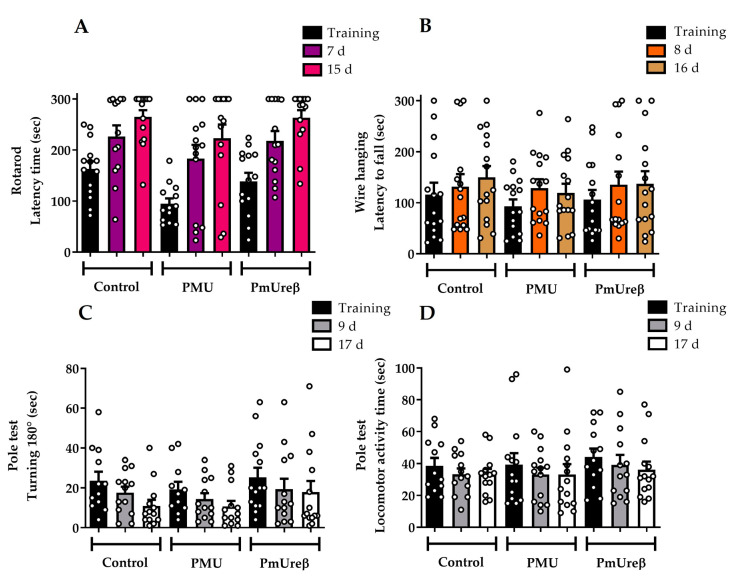
Motor tests. The animals underwent training sessions one day before the treatments. Three sessions (maximum 5 min) were performed on each experimental day for each task, with 1 h breaks between sessions. Rotarod: On the 7th and 15th days after the last injection, the animals were placed on a rotating platform and challenged to remain stable on the axis. The time taken for the animal to fall was recorded. All the groups significantly improved their task performance across the days. No effect of treatment was observed (**A**). Wire hanging box: On the 8th and 16th days after the last injection, the mice were placed on a grid and immediately turned upside down. The time it took for the animal to fall was recorded. All the groups showed a grip force in the normal range, and no treatment effect was detected (**B**). Pole test: On the 9th and 17th days after the last injection, the animals were placed on top of a pole and evaluated for the time taken to turn toward the ground (180°) (**C**) and to reach the pole base (locomotor activity) (**D**). All the groups improved their performance across the test days, and no treatment effect was observed. The analyses were performed via two-way ANOVA for repeated measures, followed by Tukey’s post hoc test. The results are expressed as the mean ± SEM (n = 15).

In the rotarod tests (Figure 2A), the analysis of the latency to fall (indicative of motor coordination and balance) did not indicate a statistically significant difference between the groups on all the test days. All the mice groups performed the same before treatment, showing that the pre-training was effective. Moreover, the PMU and PmUreβ did not impair motor coordination, since the performance of all the groups continued to improve across the days.

For the wire hanging test, the time elapsed until the animal fell off the grid (latency to fall, muscle resistance analysis) was recorded (Figure 2B). No statistically significant difference was observed between the groups at the baseline evaluation, and their performance remained similar after the administration of the protein. Thus, we can assume that the PMU and PmUreβ did not change the muscular strength.

At last, the pole test was performed on days 9 and 17 after administration. The animals were placed on the top of the pole facing upward. In Figure 2C,D, we can observe that all the experimental groups improved their performance in the tasks across the days, since the latency to turn and to move down on the pole reduced compared to the baseline. Once more, no statistically significant differences were observed between the groups.

#### 3.2.2. Open Field Test

**Figure 3:**
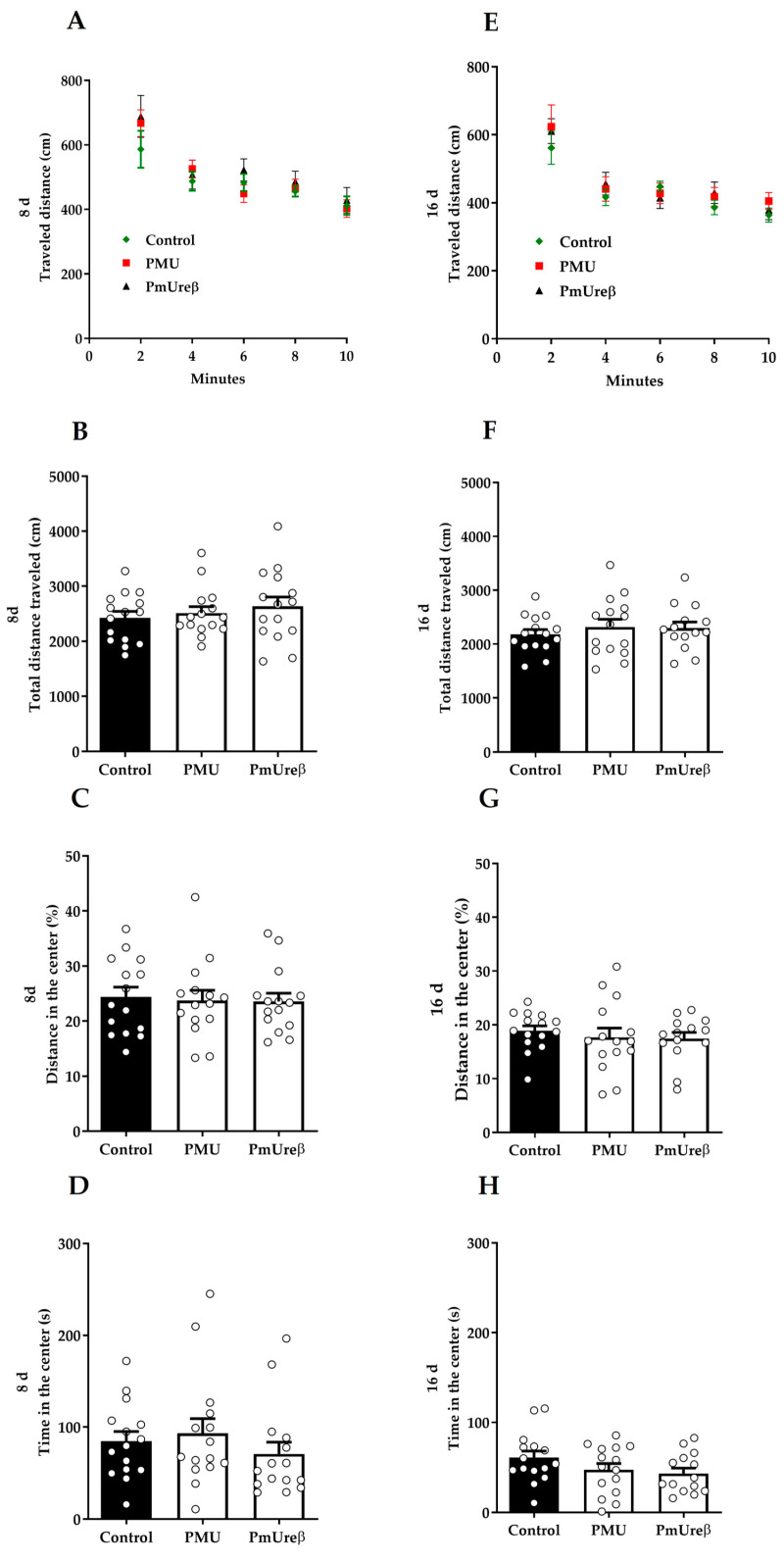
Open field test. On the 8th and 16th days after the last injection, the animals were placed in the center of an open field and left for 10 min to explore it. The total distance covered across time (cm) (**A**,**E**) showed that all the groups successfully habituated to the task. The total distance covered (cm) (**B**,**F**), percentage of distance at the center of the box (**C**,**G**), and time at the center of the box (s) (**D**,**H**) were calculated using the *MouseGlob* v. 1.0 free software. No treatment effect was observed in terms of the mice’s locomotion and thigmotaxis. The analyses were performed via two-way ANOVA for repeated measures (**A**,**E**) or one-way ANOVA (**B**–**D**,**F**–**H**), followed by Tukey’s post hoc test. The results are expressed as the mean ± SEM (n = 15).

The open field test assesses the animals’ general locomotor activity and willingness to explore a new environment by computing the animals’ traveled distance in the field. Figure 3A,E show the locomotion profile over time, indicating that all the experimental groups successfully habituated to the new environment. In addition, no significant differences could be observed between the groups, which is confirmed by the total traveled distance data (Figure 3B,F). Thus, this result is in line with data from the more specific motor assessments (Figure 2), confirming that neither of the proteins affected the mice’s locomotion.

Besides locomotion, thigmotaxis parameters can also be assessed during the open field task as a preliminary evaluation of the animal’s anxiety level. For example, an increase in the distance traveled in the center of the field (Figure 3C,G) or in the time spent in this same area (Figure 3D,H) might indicate a lower anxiety background or an anxiolytic effect [71,72]. As expected, all the groups showed a greater preference to stay close to the field walls, and no statistically significant difference was detected between the groups.

#### 3.2.3. Anxiety Tests: Elevated Plus Maze and Dark–Light Box

**Figure 4:**
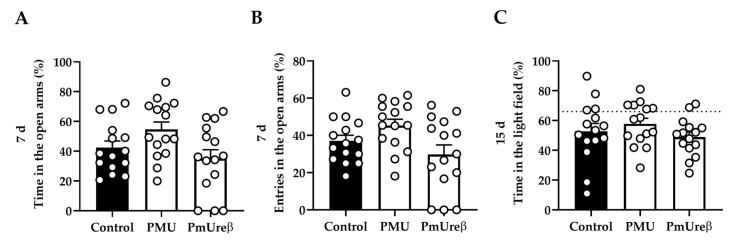
Anxiety-like behavior. Elevated plus maze: On the 7th day after the end of the administrations, the animals were submitted to the elevated plus maze behavioral test for 5 min. The animals were evaluated for the percentage of time the animal remained in the open arm (**A**) and the percentage of entries in open arm (**B**). The analyses were performed via parametric one-way ANOVA with Tukey’s post hoc test. The results are expressed as the mean ± SEM (n = 15 animals for each group). Dark–light box: On the 15th day after the end of the administrations, the animals were submitted to the dark–light box behavioral test for 5 min The animals were evaluated using the *MouseGlob* v. 1.0 software in order to determine the time in the light field (**C**). The analyses were performed via parametric one-way ANOVA with Tukey’s post hoc test. The results are expressed as the mean ± SEM (n = 15 animals for each group).

The elevated plus maze (EPM) and dark–light box (DLB) tests were chosen to evaluate anxiety-like behavior because rodents have a natural aversion to open spaces, heights, and bright environments [63,64]. Thus, in these tests, animals face a conflict between their curiosity to explore new spaces and the aversive nature of those spaces, generating anxiety. The EPM test was performed on the 7th day after the last injection. The evaluation of the percentage of permanence time and the percentual number of entries in the aversive open arms reflects an animal’s anxiety state (the longer the permanence and entries, the less anxious the animal is) [63]. Our results showed a significant difference between the PMU and PmUreβ groups, as the PmUreβ-exposed mice entered fewer times (*p* = 0.020) and spent less time (*p* = 0.026) in the open arms. However, no significant difference was observed for the treated animals when compared to the controls (Figure 4A,B). No significant difference between the groups was observed for the number of risk analysis behaviors and the total number of entries in the arms.

In order to not repeat the same anxiety test used on the 7th day, the dark–light box test was performed on the 15th day after the injections. In this test, the longer the animal stays in the bright field, the less anxious it is [64]. Our results indicated no statistically significant differences that could signal changes in the level of anxiety in the protein-treated groups as compared to the controls, since all the experimental groups traveled the same percentage of distance in the light part of the box (*p* = 0.332, Figure 4C). No significant differences between the groups were observed in the number of crossings.

#### 3.2.4. Tail Suspension Test

**Figure 5:**
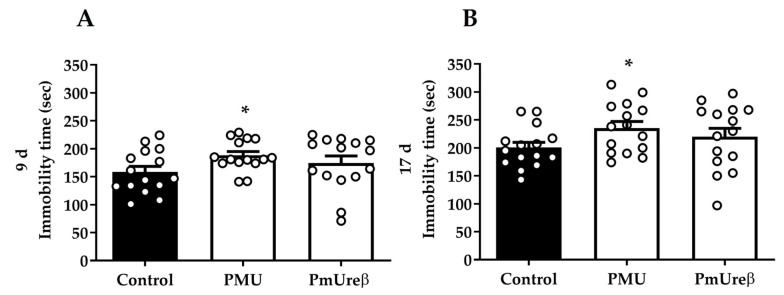
Depressive-like behavior: On the 9th (**A**) and 17th (**B**) days after the last injection, the animals were challenged in the tail suspension test. The mice were hung by their tail for a maximum of 6 min. A significant increase in the immobility time of the PMU group was observed in both task sessions, indicating depressive-like behavior in these animals. The analyses were performed by one-way ANOVA followed by Tukey’s post hoc test. The results are expressed as the mean ± SEM (n = 15). * *p* < 0.05.

The tail suspension test is a behavioral test widely used to assess the response to acutely stressful situations, usually related to apathy and depression-like behaviors [73]. The mice were subjected to the tail suspension test on the 9th (Figure 5A) and the 17th (Figure 5B) days after the protein administration. In both sessions, the mice injected with PMU showed an average increase in the immobility time (*p* < 0.05) compared to control mice. Although not statistically significant, the PmUreβ-treated mice showed a tendency to increased immobility time, as a trend toward a depressive-like state. Our results indicate that repeated injections of PMU for one week induced a long-lasting depressive-like state.

### 3.3. Western Blot Analyses 

**Figure 6:**
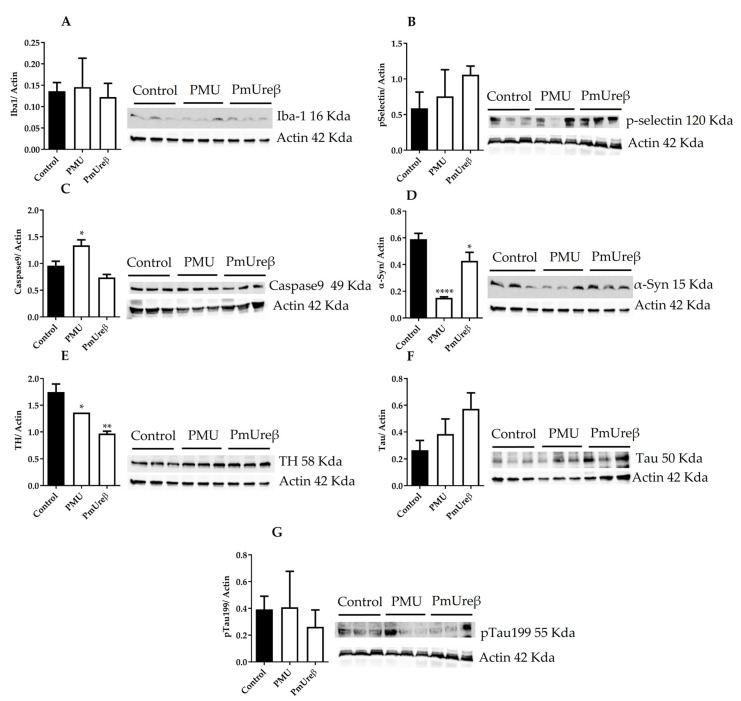
Neuroinflammation and pathological markers in the brain homogenates of treated mice. The animals received i.p. injections of 20 µg/animal/day of PMU, PmUreβ or sterile PBS (control) for 7 consecutive days and were euthanized on the 18th day after the last injection. Western blot assays were performed in the whole brain homogenates of at least three animals from each group, using 20 µg of tissue protein per lane. The levels of immunoreactive proteins were quantified via densitometry and normalized using those of actin. The images show representative blots for: Iba 1 (**A**), p-selectin (**B**), caspase-9 (**C**), α-synuclein (**D**), tyrosine hydroxylase (**E**), tau protein (**F**), and tau protein phosphorylated at Ser199 (**G**). The analyses were performed with one-way ANOVA followed by Tukey’s post hoc test. The results are the mean ± SEM (n = 3) * *p* < 0.05, ** *p* < 0.01, **** *p* < 0.0001 vs. controls.

Mice from all the groups were euthanized on the 18th day after the last injection, their brains were dissected, and the brain homogenates (supernatants) were submitted to Western blot analyses. Microglial activation was evaluated through the protein levels of Iba-1 [46] (Figure 6A) and p-selectin (mediator of microglia proliferation) [74] (Figure 6B), with no statistically significant difference among the groups. 

The accumulation of α-synuclein and the death of dopaminergic neurons are hallmarks of Parkinson’s disease that culminate in motor deficits [19]. Western blot assays of the brain homogenates of the treated mice revealed the activation of the caspase 9 (Figure 6C) and a reduction in the levels of tyrosine hydrolase (Figure 6E), a marker of dopaminergic neurons, suggesting the activation of cell death pathway(s), both in the PMU- and PmUreβ-treated animals. The content of α-synuclein (soluble form present in the homogenates’ supernatants) decreased significantly in both treated groups, with the PMU causing a reduction to about 1/3 of the level found in the control mice (Figure 6D). The accumulation of hyperphosphorylated tau protein leads to neuronal death in some neurodegenerative pathologies, such as Alzheimer’s disease [75]. The analysis of the total content of tau (Figure 6F) and of hyperphosphorylated (Ser199) tau (Figure 6G) in the brain homogenates did not show any statistical difference among the groups. 

Except for the activated caspase-9, the same trend was observed for the analyzed proteins in the brain homogenates of the PMU- and PmUreβ-treated animals. Our findings indicate that animals treated with ureases probably undergo a decrease in the population of dopaminergic neurons, which could ultimately lead to a Parkinson’s-like pathology. 

### 3.4. Integrity of the Blood–Brain Barrier

**Figure 7:**
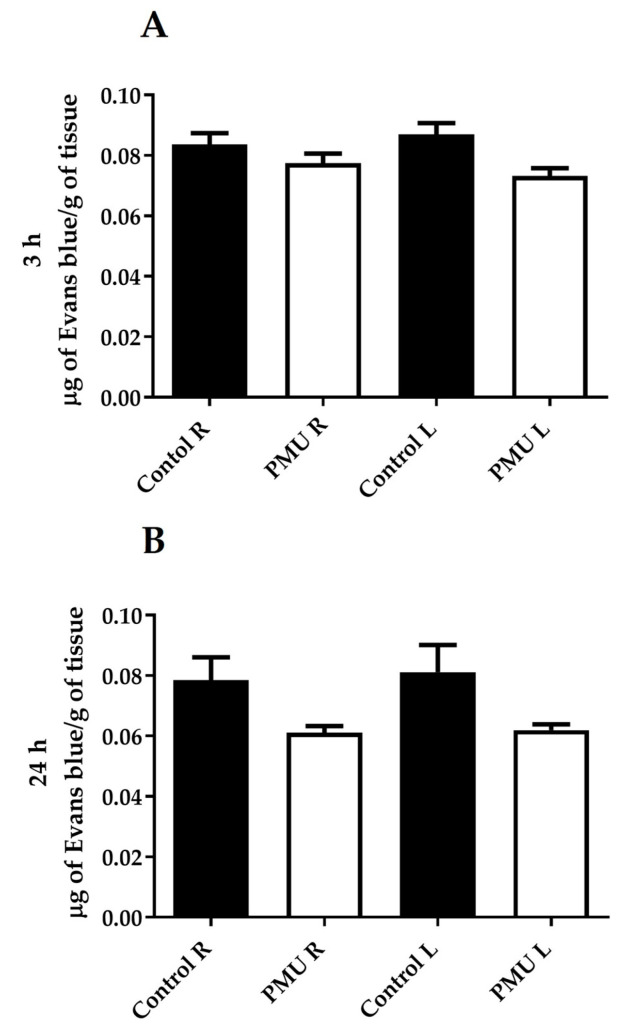

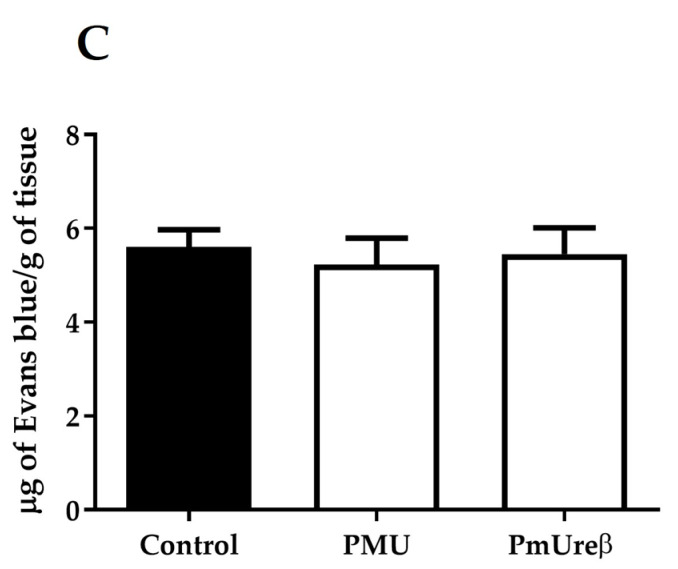
Permeability of the blood–brain barrier. The Balb/C mice were injected (e.v.) once with 400 μg PMU/animal (20 mg/kg), and 4 h later, the animals received (i.p.) a solution of 2% Evans blue (0.01 mL/100 g body weight). After 3 h (**A**) and 24 h (**B**), the mice were euthanized, their brains were dissected, and the right (R) and left (L) hemispheres were separated. The tissues were homogenized, diluted in acetic acid, centrifuged, and the absorbance of the supernatants was read at 610 nm. The data (means ± SEM, n = 5 for control, 6 for treatments) were analyzed via one-way ANOVA followed by Tukey’s post hoc test. (**C**). The Swiss mice were treated daily with (i.p.) injections with 20 µg of PMU or PmUreβ for 7 consecutive days. On the 18th day after the last injection, the animals received (i.p.) a solution of 2% Evans blue (0.01 mL/100 g body weight). After 24 h, the animals were euthanized, and their brains were collected, homogenized, and processed as described above. The amount of Evans blue present in the brain homogenates was estimated via the absorbance at 610 nm. The results are the means ± SEM (n = 5 per group). The analyses were performed via one-way ANOVA with Tukey’s post hoc test.

The endovenous administration of the PMU (20 mg/kg) into the mice did not acutely (after 3 or 24 h) induce damage to the BBB (Figure 7A,B). Nor were any alterations in the BBB detected in the animals 18 days after 7-day (i.p., 20 μg protein/animal/day) treatment with either PMU or PmUreβ (Figure 7C).

### 3.5. Effects of PMU in Cell Cultures

As mentioned earlier, we have previously demonstrated the non-enzymatic effects of PMU in cultured cells, which occurred despite there being no increase in the ammonia levels in the medium [13]. Incubation with nanomolar concentrations of PMU for up to 6 h increased the levels of pro-inflammatory cytokines (TNF-α and IL-1B) in cultured human renal cells (Hek 293) and in murine microglia (BV-2) and induced the production of reactive oxygen species in Hek 293 and SH-SY5Y (human neuroblastoma) cells [13]. 

**Figure 8:**
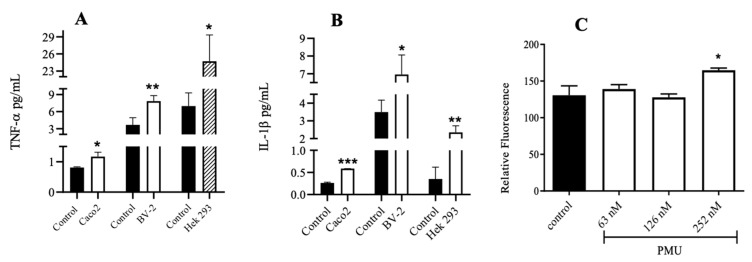
Effect of *P. mirabilis* urease (PMU) on cell lines. The cell cultures were incubated with NaPB 7.5 (control) or PMU (63, 126, and 252 nM) at 37 °C for 6 h. The production of cytokines was measured in the culture’s supernatant via ELISA. In (**A**), the TNF-α levels induced by PMU 63 nM (white bars) or 126 nM (hatched bar). In (**B**), the IL-1β levels induced by PMU 252 nM (white bars). The results are expressed in pg/mL. In panel (**C**), Hek 293 cells grown in Transwell inserts were treated for 1 h with PMU, then the medium was changed to a solution containing FITC-Dextran (1 mg/mL). After 30 min, the presence of FITC-Dextran in the lower compartment was evaluated fluorometrically (495 nm/530 nm). The results are expressed as the FITC-Dextran relative fluorescence intensity. The analyses were performed via one-way ANOVA followed by Tukey’s post hoc test. The results are expressed as the mean ± SEM (n = 3–4, per group). * *p* < 0.05, ** *p* < 0.01 and *** *p* < 0.001 vs. controls. Data on the cytokine levels in the Hek 293 and BV-2 cells were adapted from Grahl et al. (2021) [13].

Here, we demonstrated that PMU can also trigger an inflammatory response in a colorectal adenocarcinoma cell line (Caco-2). After 6 h of incubation with PMU, a significant increase in the TNF-α and IL-1β levels was observed in these cells (Figure 8A,B).

### 3.6. Paracellular Permeability of Cell Monolayers

Consistent with a pro-inflammatory effect, our data reveal that PMU can damage the cellular junctions of a monolayer of Hek 293 cells, as indicated by the passage of FITC-Dextran, after 1 h of incubation with PMU at 252 nM (Figure 8C). 

### 3.7. Morphology of α-Synuclein Aggregates Formed in the Presence of Ureases

**Figure 9:**
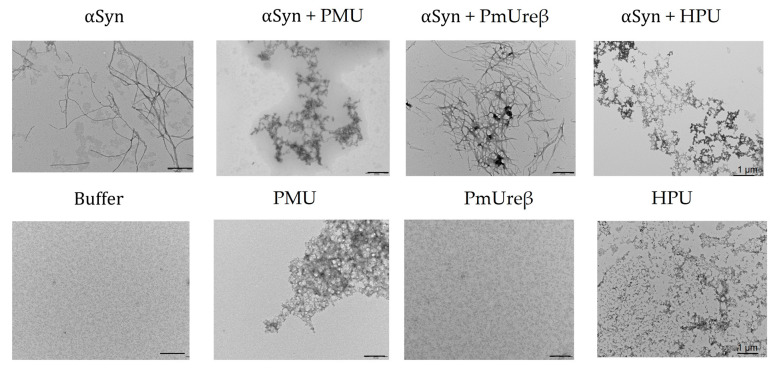
TEM images of α-synuclein aggregates formed in the presence of ureases. The reaction mixtures contained 50 µM α-synuclein in the absence (**bottom** panels) or the presence (**top** panels) of 5 µM ureases (PMU, PmUreβ, or HPU) in 10 mM NaPB, pH 7.5, 100 mM NaCl (buffer). Incubation proceeded for 150 h under gentle agitation at 37 °C. Fibril formation was confirmed via transmission electron microscopy of the reaction mixtures. The microscopies are typical results of at least three different samples. Scales bar represents 500 nm, with the exception of αSyn + HPU and HPU (1 μm).

The fibrillation of α-synuclein triggers the formation of Lewy bodies and affects dopaminergic neurons, which are believed to be related to the characteristic motor deficits in Parkinson’s disease [76]. In this work, we evaluated the effect of PMU, PmUreβ or *H. pylori* urease (HPU) on the morphology of α-synuclein aggregates. Ureases or PmUreβ were added to purified α-synuclein samples, and the mixtures were kept under gentle agitation at 37 °C for ~150 h. Our findings indicate that PMU as well as HPU led to the formation of α-synuclein aggregates with morphologies quite distinct from those formed by α-synuclein incubated alone, as indicated by the TEM analysis (Figure 9). Only a fragmented structure (amorphous aggregates and/or oligomeric clusters) was observed in the presence of urease, while α-synuclein alone exhibited long and straight classical filamentous aggregates (fibrils). In contrast, the incubation of PmUreβ with α-synuclein did not alter the apparent structure of the fibril formed. Incubation of the ureases alone also did not form fibril-like aggregates. 

## 4. Discussion

Neurodegenerative disorders, such as Alzheimer’s disease (AD) or Parkinson’s disease (PD), are accompanied by the loss of specific neurons and alterations in the conformation of proteins such as β-amyloid and tau (in AD) and α-synuclein (in PD). Changes in the conformation and/or oligomeric state of proteins and neuroinflammation may be present well before the onset of clinical symptoms, characterizing the prodromal phase of these diseases [14,77,78,79,80,81]. In the pathogenesis of PD, changes in the α-synuclein conformation and oligomeric states (monomers, fibrils, or oligomers) lead to inflammatory responses and neuronal dysfunction [82,83,84,85,86,87,88,89,90]. The altered form(s) of α-synuclein contributes to the death of dopaminergic neurons, culminating in motor symptoms [91]. 

Alterations in gut–brain communication are relevant in CNS disorders such as anxiety [92], major depression [93], autism spectrum disorders [94], AD [40,95] and PD [96]. For instance, infection with the gastric pathogen *H. pylori* has been positively correlated with PD’s progression [97,98]. Epidemiologic data show that PD patients are 1.5–3-fold more likely to have *H. pylori* infection than people without PD [98]. *H. pylori*-infected PD patients display worse motor functions than *H. pylori*-negative patients [99], and eradicating *H. pylori* improves motor function and levodopa absorption in PD patients [100].

Changes in the intestinal microbiota can be a risk factor for proteinopathies, such as PD [96,101,102,103]. Gastrointestinal disorders and constipation are common symptoms that precede motor deficits in PD patients, suggesting a cause–effect relationship between gut dysbiosis and PD’s pathophysiology [104,105,106,107,108,109]. In PD, gut dysbiosis is characterized by a change in the prevalence of *Enterobacteriaceae*, among which is *P. mirabilis* [26]. The presence of *Proteus* in the gut correlates with the severity of the disease [100], and antibiotic treatments that decrease this bacterial population ameliorate the symptoms of PD [110].

Experimental evidence in animal models reinforced a link between gastrointestinal bacteria and neurodegenerative diseases. Wang and co-workers reported that a “filtrate” of *H. pylori* culture given i.p. to rats for 7 days led to an increase in the brain levels of β-amyloid (1–42) and hyperphosphorylated tau, which are typical of AD. Moreover, the animals presented impaired spatial learning and memory [111,112]. In the case of PD and *Proteus*, Choi and co-workers employed a suspension of *P. mirabilis* dosed daily to mice via the oral route for 7 consecutive days. On the 8th and 16th days after the last administration, the animals were subjected to behavioral tests, revealing significant motor alterations, similar to a PD-like pathology. The loss of dopaminergic neurons in the substantia nigra and striatum and deposits of α-synuclein were observed in the brain and colon. The brain homogenates of treated mice showed increased levels of pro-inflammatory markers (Iba1, TNF-α, and IL-1β) [18].

The hypothesis that bacterial ureases, particularly HPU and PMU, could somehow contribute to the role that *H. pylori* and *P. mirabilis* putatively have in neurodegenerative diseases came from the observation that ureases can be highly neurotoxic. Canatoxin and JBU (jack bean urease) are urease isoforms produced by the legume *Canavalia ensiformis* (jack bean) [49]. Both ureases are highly neurotoxic, with LD_50_ 2 mg/kg (i.p. or e.v.) in rodents, and induce tonic–clonic seizures preceding death [48]. The pro-convulsant and lethal effect of canatoxin invariably occurs within 24 h of its administration, and at higher doses, seizures occur after 15–20 min [48]. Other symptoms induced by canatoxin include bradycardia, hypotension, hypothermia, hypoxia, and biphasic alterations of glycemia [48,57,113]. Studies involving canatoxin and JBU in rodents revealed an electroencephalographic pattern and brain imaging consistent with a seizure-inducing effect and alterations in synaptic plasticity, with persistent long-term depression in the hippocampus [55]. In vitro, *C. ensiformis* ureases induced L-glutamate release, raised the intracellular levels of calcium, and increased the rate of spontaneous firing of neurons [55]. Canatoxin did not affect the voltage-gated Na^+^, K^+^, or Ca^2+^ currents, nor did it interfere with the cholinergic receptors [55], suggesting an indirect mode of action possibly related to membrane-disturbing properties [114,115]. Seizures and hypothermia were also described as symptoms that precede the death of rats injected intraperitoneally with purified *H. pylori* urease [116].

Many of the biological effects of canatoxin and JBU, including their seizure-inducing and lethal activities, were found to be independent of ureolysis [49]. One of the goals of this work was to perform structure versus activity studies of PMU and to elucidate which effects could be independent of its enzymatic action. To demonstrate that biological effects do not require the enzyme activity of ureases, different experimental approaches have been used: irreversible inhibitors of the ureolytic active site [45,49,117], enzyme-incompetent (lack of nickel in the active site) apourease [118] and urease-derived polypeptides, either produced by proteolysis [119,120] or, in the case of multi-chain ureases, studies were conducted with isolated subunits [43,50]. Bearing in mind that the toxic effects of PMU could be unrelated to its enzyme activity, we compared some of the effects of the holoprotein PMU with those of its isolated B subunit (PmUreβ), which is devoid of ureolytic activity. We have previously demonstrated that PmUreβ holds some of the toxic effects of the holoprotein, such as the ability to activate blood platelets and insecticidal properties [50].

Initially, the mice and rats were injected i.p. or e.v. with PMU at doses as high as 20 mg/kg and followed up to 48 h. Surprisingly, no seizures or death were observed, contrasting with the effects seen in rodents injected with 10-fold lower doses of *C. ensiformis* or *H. pylori* ureases. The PMU-treated mice showed hyperglycemia (Figure 1), an effect also described for canatoxin-treated rats (0.5 LD_50_ or 1 mg/kg, i.p.), which induced a short lasting (1 h) increase in the blood glucose levels, followed by a hypoglycemic response with hyperinsulinemia [57,113]. There was no change in the body temperature of the PMU-treated animals, contrasting with the hypothermia seen in canatoxin-treated [48] as well as in HPU-treated mice [116]. 

The reasons for the lack of acute (within 48 h after injection) effects in the rodents treated with PMU as compared to canatoxin or HPU are not clear. Although sharing about 62% identity in their amino acids sequences, the proteins differ in the quaternary structure and native molecular mass: canatoxin, with 270 kDa, is a trimer of 90 kDa subunits [121]; HPU, with 1.06 MDa, is dodecamer of two subunits, A (30 kDa) and B (62 kDa); while PMU, with 252 kDa, is a trimer of three subunits, A or γ (11 kDa), B or β (12.2 kDa) and C or α (61 kDa). Considering the native molecular mass, the biodistributions of canatoxin and PMU are probably similar and faster than that of HPU. Thus, the differences in molecular size do not explain PMU’s lack of acute effects.

Here, an enzymatically active PMU was used for all the biological tests. Although the toxicity of *C. ensiformis* ureases does not depend on the enzymes’ activity [49], it is plausible that the hyperammonemia generated by the systemic administration of an active urease could synergize with the non-enzymatic effects of the protein. However, the kinetic parameters of PMU’s ureolytic activity are very similar to those of canatoxin and HPU, meaning they probably induce the same level of hyperammonemia. The three ureases have a similar K_M_ for urea (2–6 mM), optimal pH (7.0–8.0), and specific activity (JBU/CNTX 2700-3500; PMU 2000; HPU 1700 μmol urea cleaved/min/μg protein, respectively) [122]. As *P. mirabilis* is a commensal microorganism present in the normal gut, one can speculate that evolutionary pressures probably “shaped” PMU, as well as other ureases produced by intestinal microbiota, to be less toxic when confined to this environment. However, when the *P. mirabilis* population increases, like in PD dysbiosis, or the bacteria grow in other tissues, the full spectrum of the ureases’ toxicity, through both enzyme-dependent and independent effects, may unravel. 

Next, we treated the mice with PMU or PmUreβ given i.p. (to avoid inactivation in the stomach) daily for 7 days, and we performed behavioral tests 8 and 16 days after the last injection. A “small” dose (20 μg/animal) was chosen considering that gut dysbiosis (and increased *P. mirabilis* population) is a chronic condition associated with PD. No motor alterations (Figure 2 and Figure 3) were observed in the treated animals. The schedule was chosen to match that Choi and co-workers (2018) employed in their study with a *P. mirabilis* suspension administered orally to mice [18]. While the production of PMU required successful gut colonization by *P. mirabilis* in the previous work, here, the protein was promptly available and susceptible to degradation, which might have made it less likely that the effects could still be seen on the 8th or 16th day after the last injection.

Nevertheless, the PMU treatment induced depressive-like behavioral changes, observed even 16 days after the last injection (Figure 5). Changes in the intestinal microbiota, with an increased prevalence of *Enterobacteriaceae*, including *P. mirabilis*, have been related to depression symptoms [93]. Anxiety and depression are an early manifestation of diseases related to the deposition of Lewy bodies in neurons [123,124].

Hyperammonemia is a cause of altered mental states, including depression and anxiety [125,126,127]. Hyperammonemia associated with *H. pylori* and particularly with urinary infections by urea-splitting bacteria may impact the cognition and behavior of patients [128,129,130]. Although not measured here, hyperammonemia could be expected in mice treated with the enzymatically active PMU. However, if present, the ammonia levels probably are only mildly increased, as no motor symptoms characteristic of hyperammonemia, such as ataxia, altered levels of consciousness, seizures, loss of appetite, vomiting, coma, or respiratory distress, were noticed [131,132,133]. In this context, it is worth mentioning that in rats injected with canatoxin or JBU, alterations in synaptic plasticity with persistent long-term depression were observed at a time that the animals showed extreme prostration [55]. Altogether, the data indicated that mice treated with low doses of PMU developed behavioral alterations, such as depression, which is a non-motor symptom known to precede neurodegenerative diseases [123,134,135,136,137,138,139]. Longer schedules of PMU treatment as well as tests in aged animals might increase the likelihood of progression of the initial stages of the neurological condition seen here to culminate in motor deficits and cognitive decline characteristic of a neurodegenerative disease.

Since peripheral inflammation contributes to neurodegenerative diseases, the pro-inflammatory effects of PMU were evaluated in a human intestinal cell line. After 6 h of incubation with PMU, increased TNF-α and IL-1β were detected in Caco-2 cell cultures (Figure 8A,B). A PMU-induced increase in pro-inflammatory cytokines was previously observed in the renal cells (Hek 293) and microglia (BV-2), while no increase in ammonia levels was seen in the medium, characterizing an enzyme-independent effect [13]. The pro-inflammatory properties of ureases have been previously characterized, such as the induction of paw edema [45,140], increased vascular permeability [47], activation of neutrophils [45], activation and conversion of blood platelets to a pro-inflammatory phenotype [43,44], production of NO, pro-inflammatory cytokines, reactive oxygen species, and up-regulation of cyclo-oxygenase(s) and lipoxygenase(s) [43,45,46,47]. The pro-inflammatory activity of HPU is mediated by eicosanoids and not blocked by polymyxin B, discarding the contribution of any contaminant LPS to the inflammatory effect [47].

Increased pro-inflammatory cytokines can trigger cytoskeleton changes and disruption of endothelial junctions, resulting in augmented cellular permeability [141,142]. Here, PMU was shown to increase the cellular permeability in the monolayers of renal epithelial cells (Hek 293) (Figure 8C), accompanying the production of TNF-α and IL-1β [13]. Urease-dependent changes in cellular permeability have been previously reported for HPU in the gastric epithelial MKN28 cell line [143] and associated with disruption of the gastric mucosal barrier [144,145]. HPU also induces changes in the permeability of human microvasculature endothelial HMEC-1 cells, accompanied by profound changes in the cytoskeleton [47]. 

Since the blood–brain barrier (BBB) is composed of endothelial cells [146], urease-induced changes in endothelial permeability could indicate that the protein may reach the CNS. Hyperammonemia, at least partially due to urease’s activity associated with bacterial infections in cases of PD or AD, contributes to changes in the BBB [131,142,147,148,149,150]. Thus, the effects of hyperammonemia generated enzymatically, and the direct action of urease in altering cellular permeability, can putatively be additive or act synergistically to damage the BBB and allow circulating ureases to enter the CNS. Here, we showed that PMU, either given as a single e.v. dose or as a 7-day i.p. treatment, apparently did not damage the BBB, as suggested by the absence of the Evans blue dye in the brain homogenates. Although Evans blue is still the most used marker of brain barrier integrity, there are several limitations to its in vivo application and quantification in brain homogenates [151]. The distribution of Evans blue in the brain is not homogenous, as the dye crosses the BBB most effectively at the prefrontal cortex and the cerebellum and poorly at the striatum [152]. Hence, any localized BBB leakage induced by PMU could have been missed by the dilution of the Evans blue in the whole brain homogenates. While no acute neurotoxic effect of PMU was seen (thus, no damage to the BBB was expected), neuroinflammation and behavioral alterations were induced by the 7-day i.p. treatment with PMU. However, the analysis of BBB integrity was performed on the 18th day after the last injection, leaving time for the eventual recovery of the damaged barrier.

There are other possibilities to consider besides damage to the BBB. Ureases could gain access to the CNS through regions where the BBB is heterogeneous, such as circumventricular organs, which present fenestrated microvessels and discontinuous junctions [153,154]. The BBB can become permeable due to immune signals generated in a dysbiotic gut [94]. Circulating isolated urease, as employed in this work, represents a minor part of all the ureases produced by *P. mirabilis* that would eventually be present in a patient. In a more realistic context, ureases are contained inside the outer membrane vesicles (OMVs) produced by Gram-negative bacteria like *H. pylori* [155,156] and *P. mirabilis*. Inside the OMVs, virulence factors and bacterial toxins are protected from proteolytic degradation [157,158]. These vesicles can cross the BBB and deliver their protein cargo into the CNS [159,160], serving as carriers to shuttle the ureases produced by *H. pylori* or *P. mirabilis* into the brain. Altogether, it is too early to rule out the hypothesis that PMU systemically dosed to rodents can pass through the BBB.

While the issue of whether PMU can reach the CNS remains an open question, the neuroinflammatory effects seen in mice treated with PMU, as well as with PmUreβ, are evident, appearing in line with the increase in pro-inflammatory cytokines (Figure 8) and ROS production observed in different cultured cells. Microglia activation occurs in neurodegenerative diseases [161,162,163], although there are contradictory findings [164]. No evidence of microglial activation or increased content of total tau and Ser199-phosphorylated tau was found in the brain homogenates of treated mice (Figure 6A,B,F,G). These results contrast with our previous findings of augmented levels of Iba-1 and phosphorylated tau found in the brain homogenates of rats treated i.p. with purified HPU [46]. In that study, the brain homogenates were prepared one day after the last injection of HPU, while in the present work, the PMU-treated mice were euthanized 18 days after the last injection. Thus, partial recovery from the PMU insult after 18 days could probably explain the different data. 

On the other hand, analysis of the brain homogenates on the 18th day after the last injection revealed the activation of caspase 9 (Figure 8C) and a significant decrease in the tyrosine hydroxylase levels upon treatment with either PMU or PmUreβ (Figure 6E), which may indicate the death of dopaminergic neurons [165]. The pro-inflammatory response (ROS, cytokines) induced by ureases is probably one of the reasons for the activation of caspase-9 [13,45]. We also found a significant decrease in the α-synuclein content in the supernatants of the brain homogenates, with a reduction to ~30% of the control levels in the PMU-treated group (Figure 6D). The levels of α-synuclein may be reduced in the serum or cerebrospinal fluid of PD patients, accompanying the loss of dopaminergic neurons [166,167]. 

Microscopy images of α-synuclein incubated with PMU or HPU, but not with PmUreβ, revealed protein aggregates with a different morphology compared with the classical amyloid fibrils produced by α-synuclein alone (Figure 9). In the presence of full-length ureases, α-synuclein formed amorphous/oligomeric species, while PmUreβ apparently did not affect the formation of fibrils. In addition, these aggregates exhibited low thioflavin-T fluorescence compared to α-synuclein alone. These data suggest that the binding of PMU or HPU to α-synuclein modulates the aggregation of the protein, probably generating off-pathway oligomers incompetent to form long and straight fibrils. Presently, it is not known which oligomeric forms of α-synuclein, if any, are produced in the presence of the ureases. Interestingly, these aggregates are similar to oligomers produced from nitrotyrosinated amyloid-β peptide, a post-translational modification reported to stabilize the soluble oligomers of amyloid-β with increased synaptotoxicity [168]. Recent evidence suggests that the soluble oligomers formed early in the fibrillation process are the most cytotoxic forms of α-synuclein [169,170,171]. If the presence of PMU or HPU could shift the aggregation process of α-synuclein toward an increase in the population of oligomers, this might impact PD pathogenesis and putatively explain the positive correlation of the high prevalence of *P. mirabilis* or *H. pylori* infection among affected patients. Further studies are required to evaluate the mechanisms underlying the effect of ureases on α-synuclein aggregation as well as the toxic properties of these structures. 

Broll et al. (2021) [50] performed structure versus activity studies with the isolated subunits of PMU and mapped to PmUreβ all the tested activities (induction of platelet aggregation, fungitoxic and insecticidal activities). Here, we compared the effects induced by PMU and PmUreβ in mice. To the best of our knowledge, this is the first study conducted in vivo with an isolated subunit of a urease. The findings regarding the in vivo neuroinflammatory effects of the proteins coincided, varying in the detected levels of the analyzed markers, except for caspase 9, which was found to be elevated only in the PMU-treated animals. The proteins did not affect the motor performance of the mice after the 7-day treatment. PMU induced a depressive-like state in the treated mice, while the animals that received PmUreβ showed a tendency toward depressive behavior (Figure 5).

It should be noted that PMU is trimeric and contains three PmUreβ subunits per molecule. On the other hand, PmUreβ has a smaller size (12.2 kDa) than PMU (252 kDa), implying that it probably has faster absorption and clearance in vivo. Interestingly, PmUreβ did not interfere with the aggregation of α-synuclein. This difference can be related to the smaller size of the isolated PmUreβ and/or to the fact that PMU offers a “surface” with three exposed PmUreβ (in its [(αβγ)_3_] native form) for interaction with α-synuclein. Since the in vivo neurotoxic effects of PMU and PmUreβ had much more similarities than differences, the results showing that PmUreβ apparently did not interact in vitro with α-synuclein need to be reevaluated. At this point, the data gathered for PmUreβ support the view that it carries the biological properties of the holoprotein PMU in mammalian models. If further confirmed, PmUreβ and homologous sequences in other ureases may represent a druggable target for the future development of anti-urease therapies.

## 5. Conclusions

The role of bacterial ureases as a virulence factor in neurodegenerative diseases has been so far ignored, mostly because their large size has been considered impeditive to their entrance into the CNS. Besides the hyperammonemia produced by ureases during bacterial infections, ureases induce other effects independently of their enzymatic action, which may trigger or aggravate several pathologies, including neurodegenerative diseases. Here, we demonstrated that the systemic administration of PMU, although it not affect motor performance, induced depressive-like behavior in mice and evidence of neuroinflammation in the brain, signs that precede diseases such as AD and PD. The PmUreβ subunit represents a biologically active domain relevant to PMU-induced effects, both in vivo and in vitro. Our findings identified ureases as virulence factors that can contribute to neuroinflammation and behavioral alterations and represent unexplored targets to expand the therapeutical arsenal that could aid in the prevention or change of the course of neurodegenerative diseases.

## Data Availability

The data presented in this study are available within the article or supplementary material.

## Supplementary Materials

The following supporting information can be downloaded at https://www.mdpi.com/article/10.3390/microorganisms11082042/s1, Figure S1: Purification of recombinant *P. mirabilis* urease; Figure S2: In vivo experimental design.
