# Supplementary figures and images for: Could the Urease of the Gut Bacterium Proteus mirabilis Play a Role in the Altered Gut–Brain Talk Associated with Parkinson’s Disease?
Source: Microorganisms. 2023 Aug 9;11(8):2042. doi: 10.3390/microorganisms11082042 (PMC10459573; doi:10.3390/microorganisms11082042)

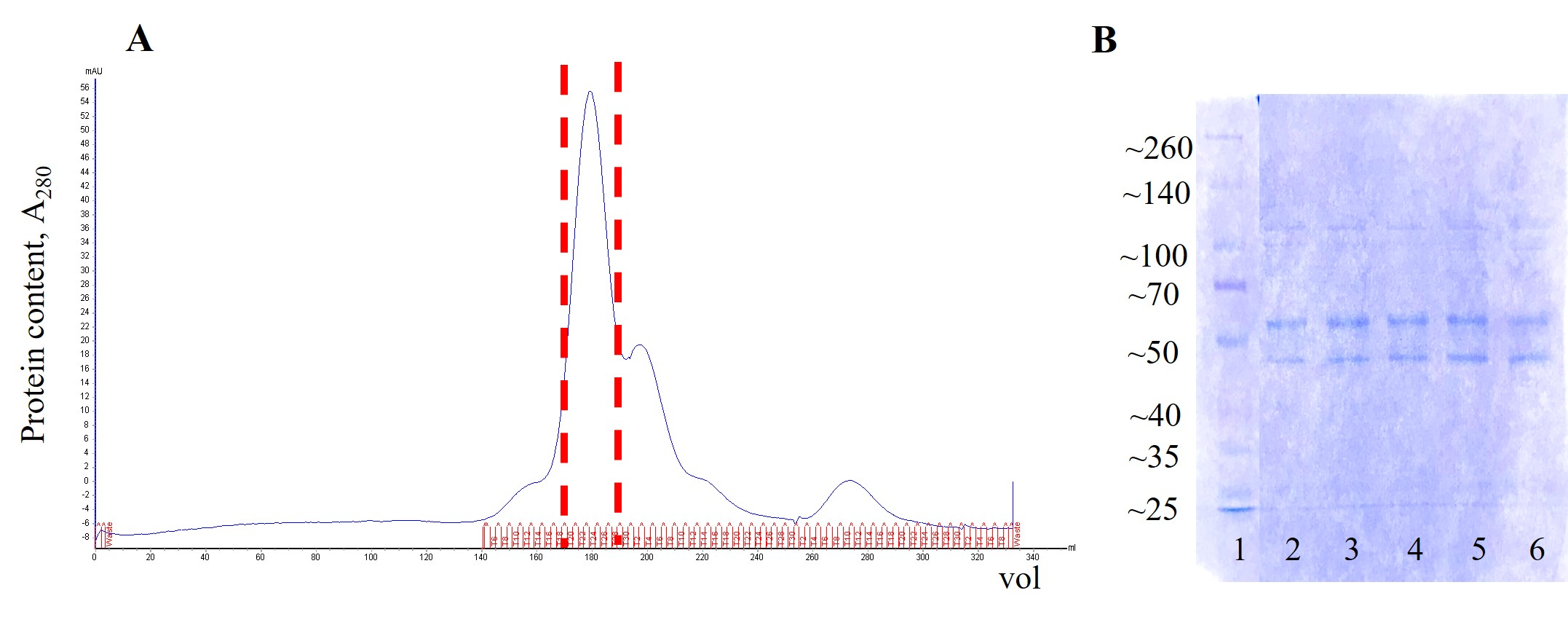

Supplement: Supplementary file 1 [file microorganisms-11-02042-s001.zip › sup Figure S1.tiff]

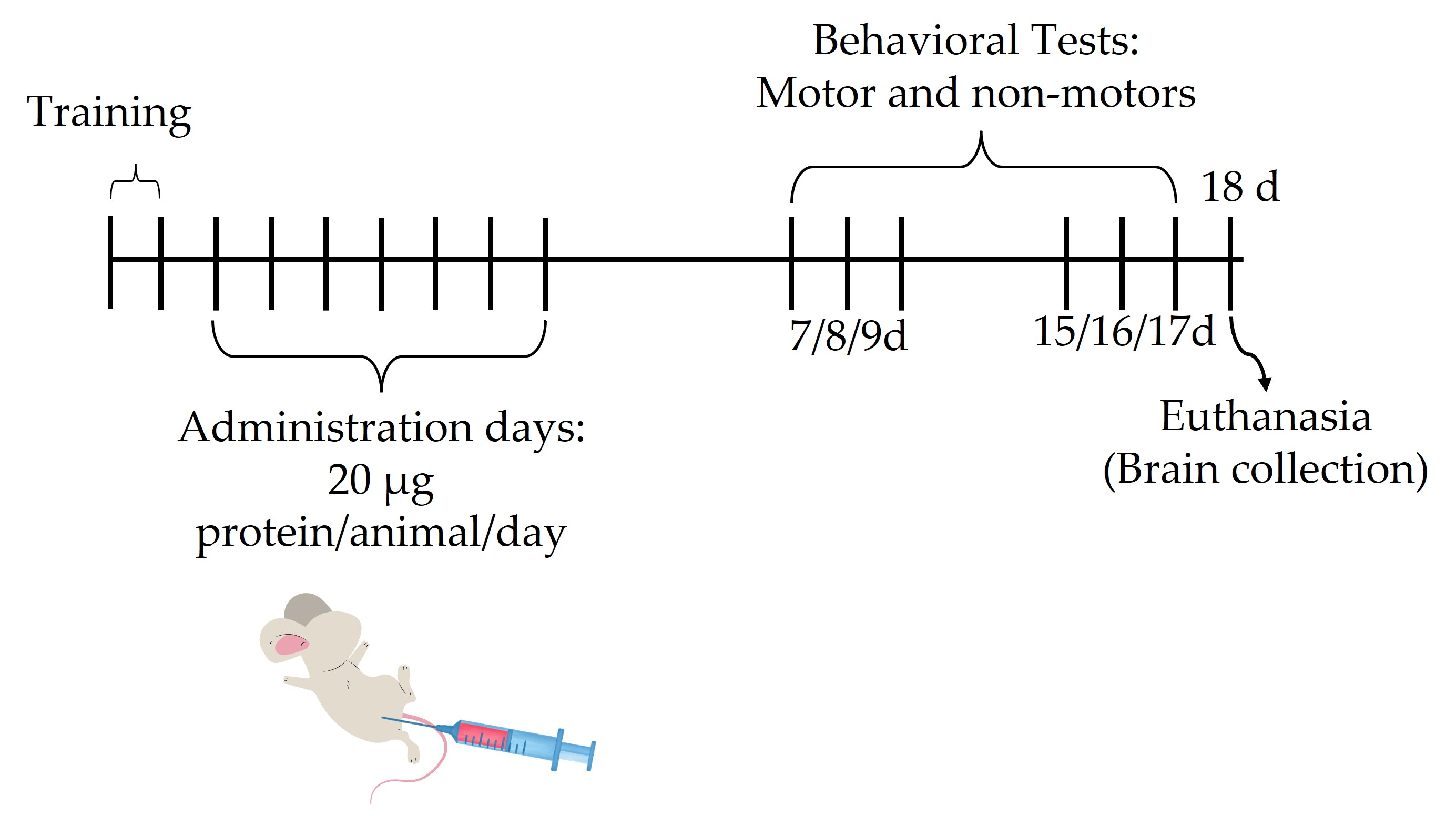

Supplement: Supplementary file 1 [file microorganisms-11-02042-s001.zip › sup Figure S2.tiff]
